# A Contemporary Report of Clinical Outcomes in Patients with Melanoma Brain Metastases

**DOI:** 10.3390/curroncol28010045

**Published:** 2021-01-13

**Authors:** William J. Phillips, Tabassom Baghai, Michael Ong, Bryan Lo, Andrea M. Ibrahim, Tyler K. T. Smith, Xinni Song

**Affiliations:** 1 Faculty of Medicine, The University of Ottawa, Ottawa, ON K1H 8L6, Canada; wphil077@uottawa.ca (W.J.P.); tsmit121@uottawa.ca (T.K.T.S.); 2 The Ottawa Hospital Research Institute, The University of Ottawa, Ottawa, ON K1H 8L6, Canada; tbaghai@ohri.ca (T.B.); mong@toh.ca (M.O.); brlo@toh.ca (B.L.); ibrahim.andrea@gmail.com (A.M.I.)

**Keywords:** Melanoma brain metastasis, immunotherapy, stereotactic radiotherapy, screening, clinical outcome, systemic therapy

## Abstract

*Background:* Brain metastases are observed in more than 40% of all patients with stage 4 melanoma. In recent years, more extensive use of stereotactic radiation (STRT) and the advent of immune checkpoint inhibitors have positively impacted outcomes in patients with metastatic melanoma.brain metastases. Here, we examined real world clinical outcomes of patients presenting with melanoma brain metastases (MBMs). *Methods:* This retrospective review evaluated MBMs patients treated at The Ottawa Hospital from April 2000 to July 2017. Clinical, radiologic, pathologic and treatment information were gathered from the electronic medical records. The primary outcome was overall survival. The proportional Cox regression model was employed for survival data, while the Fisher’s exact and Mann–Whitney U tests analyzed the relationship between categorical and continuous data, respectively. *Results:* This retrospective study included 276 patients. Brain metastases were detected symptomatically in 191 patients (69.2%); the rates of detection by routine screening were 4.6% in the pre-2012 era and 11.7% in the contemporary era (*p* = 0.029). Median survival was three months. Predictors of overall survival were age, higher lactate dehydrogenase (LDH) values, multiple brain lesions, more extensive extracranial disease, neurological symptoms, infratentorial lesions and treatment type. Multivariable analysis demonstrated that stereotactic radiotherapy (STRT) was associated with a hazard ratio of 0.401 (*p* < 0.001) for survival; likewise, immune checkpoint inhibitor therapy was associated with a hazard ratio of 0.375 (*p* < 0.001). *Conclusion:* The findings from this study as “real world” data are consistent with results of pivotal clinical trials in MBMs patients and support contemporary locoregional and immunotherapy practices.

## 1. Introduction

Melanoma brain metastases (MBMs) are common in patients with metastatic melanoma. It is estimated that 15% of patients with stage 3 melanoma will develop MBMs over the course of their illness [1]. This rate climbs higher with autopsy data; approximately 75% of stage 4 patients have evidence of MBMs at the time of autopsy [2]. Brain metastases frequently cause significant neurological complications, leading to morbidity and mortality in this patient population. Historically, the reported median overall survival of patients with MBMs is approximately four months [3]. Treatment of MBMs is challenging and requires a multidisciplinary approach. Prior to the current era of effective systemic therapies in melanoma (i.e., immune checkpoint inhibitors and BRAF/MEK-targeted therapies), the mainstay of treatment for MBMs involved a combination of locoregional modalities such as surgery, stereotactic radiation therapy (STRT) and whole brain radiation therapy (WBRT). Systemic chemotherapies provide minimal benefit as treatment for brain metastasis [4]. While temozolomide was previously used, given its ability to cross the blood–brain barrier, its performance in clinical trials was limited (5% response rate; 29% stable disease rate) [5].

In the past decade, the advent of immune checkpoint inhibitors (anti-PD-1 and anti-CTLA-4) and molecular-targeted therapies (BRAF and MEK inhibitors) has significantly improved clinical outcomes and overall survival of patients with metastatic melanoma [6,7,8,9]. However, the majority of the pivotal clinical trials investigating these agents in the setting of metastatic melanoma exclude patients with the presence of untreated brain metastases [10,11,12]. Recent studies evaluating the impact of systemic immune therapy in patients with untreated MBMs have demonstrated intracranial responses up to 50%, particularly in patients with untreated asymptomatic brain metastases not requiring corticosteroids [8,13]. These results are practice-changing, suggesting that early systemic treatment of brain metastases and routine surveillance imaging of the brain are crucial to optimize patient outcomes.

Along with recent advances in systemic therapy, the increased use of STRT has also impacted patient care. Traditionally, STRT was reserved for patients with smaller and oligo brain lesions; patients with a solitary brain lesion, greater than 3 cm in maximal diameter, were selected for surgery, while patients with more than three lesions were offered WBRT instead [14,15]. There is emerging evidence for expanding the usage of STRT, such as lower rates of neurocognitive side effects and non-inferior effectiveness compared to WBRT in patients with multiple lesions [16]. With our current therapeutic options for treating metastatic melanoma, the optimal use of combined treatment approaches warrants further evaluation. 

In this explorative, retrospective study, we audited patients diagnosed with MBMs prior to and after the era of immune checkpoint inhibitors, and evaluated how contemporary practices in locoregional and systemic therapy have impacted patient clinical outcomes. 

## 2. Methods

### 2.1. Study Design

This study is a single center, retrospective review of the survival outcomes of patients with MBMs. The aim of the study is to identify clinical factors, tumor characteristics and treatment modalities that may impact patients’ overall survival and be of prognostic value for clinicians. This study was approved by the Ottawa Health Science Network Research Ethics Board (OHSN-REB) with protocol number 20170932-01H. Patient informed consent was not required by ethics due to the retrospective nature of the study.

### 2.2. Patient Selection

Patients diagnosed with MBMs that were seen in consultation and/or who received treatment at The Ottawa Hospital Cancer Centre (TOHCC) between April 2000 and July 2017 were identified and included in this study. A diagnosis of MBMs was made on the basis of the following criteria: (1) imaging evidence of intracranial metastases (CT head or MRI head, with or without contrast) and either (2) a previous pathological diagnosis of melanoma or (3) pathological confirmation of a brain metastasis, metastatic focus or the primary melanoma. There were 281 patients that met this criterion. Five were excluded on the basis of inadequate follow up. 

### 2.3. Data Collection

Patient demographic information, primary tumor characteristics, sites of extracranial recurrences (if present) and brain metastases characteristics were collected from the hospital electronic medical records (EMR). Radiological and pathological characteristics of the lesions were obtained from radiological and pathological reports, respectively. If both CT and MRI reports were available at the time of diagnosis, MRI reports were preferred. Lactate dehydrogenase (LDH) laboratory values were considered if they were collected within 3 months of the date of brain metastases diagnosis.

The contemporary era of MBMs treatment was defined as treatment beginning in 2012, as ipilimumab was first made available for the treatment of metastatic melanoma in Ontario and was recommended in Cancer Care Ontario guidelines in 2012. Prior to then, access to immune checkpoint inhibitors was limited to use in clinical trials or through special access programs. 

Systemic therapy and radiotherapy information were determined based on the electronic systemic therapy records and physician clinical notes on the EMR. Immunotherapies consisted of PD-1 inhibitors and CTLA-4 inhibitors; targeted therapies consisted of BRAF and MEK inhibitors; and chemotherapy consisted of any cytotoxic chemotherapy regimens. Radiotherapy was classified as stereotactic radiotherapy or whole brain radiotherapy. Operative reports were retrieved to confirm and determine details of surgical intervention. 

### 2.4. Patient Outcomes

The primary outcome of this study was overall survival. Mortality was inferred based on the presence of a death certificate, discharge to a terminal care facility without follow up at TOHCC, discharge for palliative care at a community hospital or loss of follow up (without transferring care). The follow-up period ended in April 2018, at which point surviving patients were censored from subsequent analyses. 

### 2.5. Statistical Analysis

Relationships between categorical variables were analyzed using the Fisher’s exact test, while continuous data were analyzed using the Mann–Whitney U test. Multivariable relationships between categorical variables were analyzed by binary logistic regression. The relationship between variables and outcome was assessed by univariable and multivariable analysis using the Cox proportional regression model. Multivariable analysis was conducted with variables that reached a significance of *p* < 0.05, to determine independent predictors of overall survival. 

Survival analysis of specific treatment regimens was performed. Overall survival was considered the primary outcome. Comparisons of overall survival were performed through the Cox proportional regression model, while comparisons of progression-free survival were performed by binary logistic regression. Only treatments received prior to the treatment in question were controlled for in the survival analysis. Missing data were replaced by the median value of the variable. Variables with greater than 5% of missing data were subject to sensitivity analysis by replacement with the variable’s minimum and maximum values. If it was found to impact the test statistic, the more conservative *p*-value was represented. 

Statistical analysis was performed on SPSS for Mac (IBM Corp. Released 2017. IBM SPSS Statistics for Windows, Version 25.0. Armonk, NY, USA). Confidence intervals were represented as 95% certain to contain the population mean. A *p*-value of <0.05 was considered the threshold of significance. 

## 3. Results

### 3.1. Sample Characteristics

Two hundred seventy-six patients met the inclusion criteria. The baseline characteristics of the population are summarized in Table 1. The median age was 61 years, and males represented 67.0% of the sample. Seventy-eight percent of patients had a prior history of primary melanoma tumor at the time of diagnosis. One hundred seventy-three (62.7%) patients were diagnosed from 2000 to 2011, while 103 (37.3%) patients were diagnosed post-2012. The median time from the diagnosis of a primary melanoma lesion to the development of brain metastases was 24 months (Interquartile range (IQR) 11–50.5 months). The median time to development of MBMs was stage-dependent: 59.5 months in stage 1, 30 months in stage 2, 20 months in stage 3, and one month in stage 4 patients.

### 3.2. Detection of Melanoma Brain Metastases

In this cohort of patients, we evaluated the initial clinical indication for brain imaging, including new neurological symptoms, routine screening, staging/restaging or non-malignancy-related indications. The majority of brain metastases were detected during work-up for new neurological symptoms (191 patients, 69.2%), followed by staging/restaging (63 patients, 22.8%), routine screening (20 patients, 7.3%) and non-malignant indications (two patients, 0.7%). The most common symptoms resulting in brain imaging were focal neurological deficits (75 patients, 39.3%), headaches (48 patients, 25.1%) and seizures or altered level of consciousness (34 patients, 12.3%). There were also 24 patients (12.6%) presenting with more than one symptom. Detection with the presence of neurological symptoms was related to hemorrhaging lesions (*p* = 0.032), larger lesions (*p* < 0.001) and decreased overall survival (hazard ratio (HR) = 1.4, confidence interval (CI) = 1.1–1.9, *p* = 0.021) by multivariable analysis. Notably, prior to 2012, the rate of brain metastases detection from neurological symptoms was 74.0% compared to 61.4% in the contemporary era (*p* = 0.026), while rates of detection from routine screening were 4.6% compared to 11.7% (*p* = 0.029), respectively. 

### 3.3. Predictors of Overall Survival

The median overall survival of this cohort was four months (IQR 2.0–9.0 months). At the end of the study period, 260 (94.2%) patients succumbed to their disease, while 16 (5.8%) patients were alive. Factors related to poor outcome were older age, high LDH values, multiple brain lesions, more extensive extracranial disease involvement, the presence of neurological symptoms, infratentorial involvement and WBRT. Factors associated with prolonged survival included treatment with immunotherapy, chemotherapy, STRT and complete resection. Multivariable analysis of significant factors revealed that the number of extracranial sites, the presence of neurological symptoms, immunotherapy, chemotherapy, STRT and full excision were independent predictors of survival. The results of univariable and multivariable analyses are provided in Table 2. 

We investigated the survival difference between past and contemporary eras of systemic therapy. The median overall survival was four months in both eras. However, on multivariable analysis, we controlled for non-treatment covariates and identified a hazard ratio of 0.778 (CI = 0.60–1.0, *p* = 0.057) associated with MBMs treated in the contemporary era. This was not statistically significant but does demonstrate a trend for improving survival in the contemporary era. 

### 3.4. Locoregional Therapy

In this cohort, 83 (31.1%) patients had a single brain metastasis, 81 (29.3%) had oligo brain metastases (i.e., ≥2 and ≤4 lesions) and 110 (39.9%) had multiple brain metastases (>4 lesions). There were 19 (6.9%) patients with leptomeningeal disease. Seventy-four (26.8%) patients underwent surgical excision of their brain metastases, whereas 267 (96.7%) received radiation therapy. Of patients who underwent surgery, 43 (15.5%) had complete resection of their intracranial metastases, while 31 (11.2%) were treated with partial resection based on the operative report and postoperative imaging. With regards to radiation therapy, 175 (63.4%) patients received WBRT alone, 37 (13.4%) received STRT alone and 55 (19.9%) received both. 

Rates of radiation therapy over time were analyzed. In the pre-2012 era, STRT rates were 23.1%, while in the contemporary era they were 50.5% (*p* < 0.001). This difference reaches statistical significance both on univariable analysis and after controlling for the number and size of brain lesions (Table 3).

**Figure 1:**
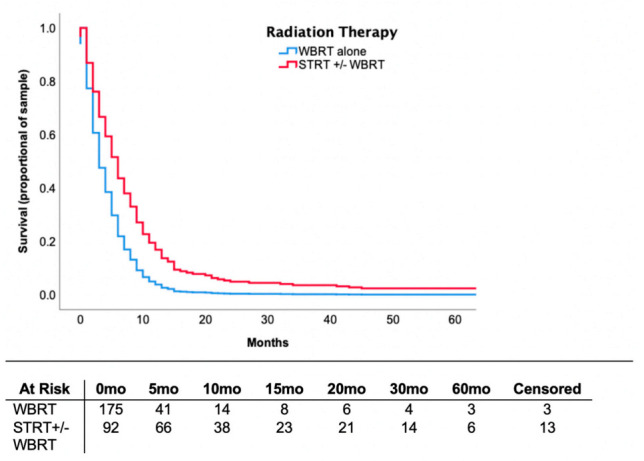
Proportional Cox regression survival curve demonstrating survival over months in patients receiving STRT ± WBRT versus WBRT alone. Hazard ratio (HR) = 0.401, 95% confidence interval (CI) = 0.294–0.548, *p* < 0.001 were associated with STRT ± WBRT compared to WBRT alone. Analysis controlled for age, LDH value, number of intracranial lesions, extent of extracranial disease, the presence of neurological symptoms, infratentorial lesions, surgery and systemic therapy. WBRT—whole brain radiation therapy; STRT—stereotactic radiation therapy.

The median overall survival of patients who received STRT was 9.0 months (IQR 5.0–15.5) compared to 3.0 months (IQR 2.0–7.0) in patients receiving WBRT alone. Controlling for patient background characteristics, including the number of brain metastases and prior treatments received, STRT was associated with an adjusted hazard ratio of 0.546 (CI = 0.40–0.74, *p* < 0.001) for mortality. Survival curve data are provided in Figure 1. At six months, 65.2% of patients receiving STRT ± WBRT survived versus 17.7% of patients receiving WBRT alone (OR = 9.5, CI = 4.4–20.5, *p* < 0.001). At two years, survival was 19.6% in the former and 2.3% in the latter (OR = 3.1, CI = 0.85–11.0, *p* = 0.086). This analysis was controlled for background variables, such as patient characteristics, imaging findings and systemic treatments received. 

### 3.5. Systemic Therapy

In this cohort, 115 (41.6%) patients received systemic therapy, 72 (26.0%) patients received chemotherapy, 44 (15.9%) received immunotherapy and 18 (6.5%) received targeted therapy. Of patients receiving immunotherapy, 18 (41.0%) were treated with CTLA-4 inhibitors, 16 (36.3%) with PD-1 inhibitors and 10 (22.7%) with doublet immunotherapy. Similarly, 11 (61.1%) patients treated with targeted therapy received a BRAF inhibitor alone, while seven (38.9%) received dual BRAF and MEK inhibitors. 

Systemic therapy use was compared between treatment eras. In the pre-2012 era, the rate of chemotherapy use was 34.1% versus 12.6% in the contemporary era (*p* < 0.001) (Table 3). Rates of targeted therapy and immunotherapy were not analyzed between eras, given their limited availability before 2012. Based on multivariable analysis of relevant clinical variables (age, gender, stage at diagnosis, number of brain lesions, extent of extracranial disease, LDH, the presence of neurological symptoms, leptomeningeal disease, location of lesions, size and the presence of hemorrhage), older age and the presence of neurological symptoms were statistically significant predictors of patients who did not receive immunotherapy use in the contemporary era. 

**Figure 2:**
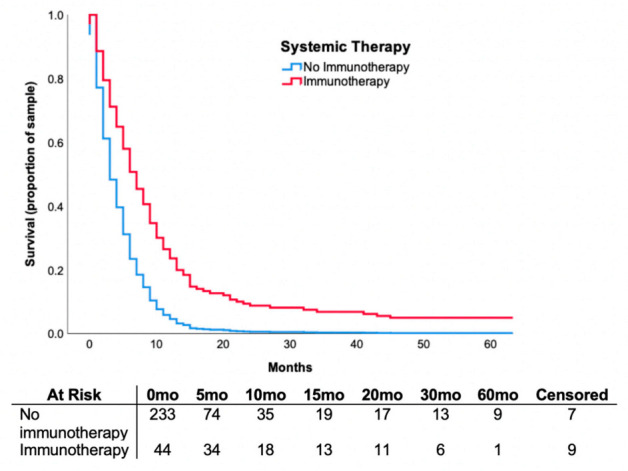
Proportional Cox regression survival curve demonstrating survival over months in patients receiving immunotherapy versus no immunotherapy. Hazard ratio (HR) = 0.375, 95% confidence interval (CI) = 0.246–0.574, *p* < 0.001 were associated with immunotherapy compared to no immunotherapy. Analysis controlled for age, LDH value, number of intracranial lesions, extent of extracranial disease, the presence of neurological symptoms, infratentorial lesions, surgery, radiation therapy type and chemotherapy.

Median overall survival was 9.0 months (IQR 6.0–19.5) in the patients who received immunotherapy, 7.5 months (IQR 5.0–11.0) in patients treated with targeted therapy, and 7.0 months (IQR 3.0–13.0) in patients treated with chemotherapy. We investigated the relationship of immunotherapy on mortality through multivariable analysis and observed that treatment was associated with a hazard ratio of 0.468 (CI = 0.31–0.70, *p* < 0.001) adjusted for confounding clinical variables, radiological findings and prior treatments. The survival curve is shown in Figure 2. When comparing patients treated with immunotherapy versus non-immunotherapy, overall survival was 68.2% versus 20.5% (OR = 6.0, CI = 2.3–15.4, *p* < 0.001) at six months and 26.3% versus 5.6% at two years (OR = 4.7, CI = 1.1–21.0, *p* = 0.044), respectively. This analysis controlled for background variables, similarly to above. 

### 3.6. Outcomes in Patients Receiving Combination of Immunotherapy and Radiotherapy

Twenty (48.8%) patients were treated with immunotherapy + STRT, 12 (29.3%) patients with immunotherapy + WBRT and 11 (26.8%) patients were treated with immunotherapy + STRT + WBRT. There were only three (7.3%) patients treated with immunotherapy without any radiation. Patients treated with immunotherapy + STRT + WBRT had a median overall survival of 15.0 months (IQR 10.0–19.5); patients treated with immunotherapy + STRT experienced a median survival of 9.0 months (IQR 7.0–29.5); and patients treated with immunotherapy + WBRT experienced a median survival of 5.5 months (IQR 4.0–9.0).

## 4. Discussion

Patients diagnosed with MBMs have worse survival outcomes compared to those without brain metastases. In our cohort of 276 patients diagnosed with MBMs between April 2000 and July 2017, the median overall survival was four months, highlighting the significant unmet need for better treatment options for patients with MBMs. Predictors of overall survival were the number of extracranial sites, the presence of neurological symptoms and treatment type, in keeping with previous reports [3,17,18,19,20,21]. 

We observed that the time to the development of brain metastases was stage-dependent. Overall, the all-stage median time to development of brain metastases was 24 months. The most common indication for brain imaging that detected brain metastases was the presence of neurological symptoms. Less than 10% of patients had brain metastases detected on routine screening and only 30% of brain metastases were detected without the presence of neurological symptoms. Our data also show that the presence of neurological symptoms is related to poorer outcomes, particularly, shortened overall survival by multivariable analysis (HR = 1.40, *p* = 0.021). With the availability of effective treatment options and evidence suggesting better outcomes in patients with asymptomatic brain metastasis, our data support routine screening imaging in the asymptomatic high-risk melanoma patient population. From this retrospective review, we also plan to do molecular profiling for patients with overall survival > 24 months and with resected brain metastases. 

### 4.1. Stereotactic Radiotherapy

STRT facilitates delivery of an accurate and high dose of radiation to a specific lesion In the context of brain metastases, STRT is mainly reserved for patients suffering with oligo brain disease (<5 brain lesions) and lesions less than 3 cm in maximal diameter. The clinical impact of STRT in this population is well-supported [14,15]. However, recent studies have investigated the utility of STRT in patients with multiple brain metastases. One group, Yamamoto et al., recently conducted a large-scale, prospective, observational study, which demonstrated that STRT alone was non-inferior to WBRT in patients with up to 5–10 brain metastases [16]. A separate retrospective analysis by Bowden et al. examined outcomes in patients with more than 15 brain lesions and found no significant difference between patients treated with STRT versus STRT + WBRT [22]. Other retrospective studies have further supported the efficacy of STRT in patients with multiple brain lesions [23,24]. These findings, in combination with reports of increased cognitive impairment in patients treated with WBRT, may explain the declining use of WBRT at our center [25]. This is particularly applicable to patients who have a robust response to systemic therapy and live long enough to experience morbidity from WBRT-related neurocognitive impairment. Current NCCN (National Comprehensive Cancer Network) guidelines recommend using STRT in combination with systemic therapy as the preferred treatment. NCCN guidelines also state that upfront WBRT is not generally recommended for MBMs and STRT is the preferred strategy if feasible. 

In this study, we observed increased use of STRT and decreased use of WBRT use in the contemporary era. The change reflects the adaptation of modern standard treatment options based on clinical evidence. The results are consistent with the findings of recent literature and demonstrate a changing paradigm in locoregional management of MBMs. 

### 4.2. Immunotherapy

Immune checkpoint inhibitors, such as anti-PD-1 and CTLA-4 agents, have greatly improved survival in patients with MBMs, as evidenced by recent prospective clinical trials [8,10,13,26]. One clinical trial examining the role of ipilimumab (CTLA-4 inhibitor) monotherapy in treatment of MBMs demonstrated a 17% objective response rate in 51 asymptomatic patients with MBMs [10]. A second trial containing 20 patients being treated with ipilimumab for MBMs demonstrated an overall survival at three years of 27.8%, which led the authors to hypothesize whether immunotherapy responders tend to have long lasting responses [11]. Similar results were achieved with anti-PD-1 agents such as pembrolizumab and nivolumab. Particularly, one phase 2 trial of 23 patients receiving pembrolizumab versus placebo reported a median overall survival of 17 months in the treatment group, with almost half (48%) of patients surviving at 24 months [12].

The CheckMate 204 study evaluated the efficacy and safety of nivolumab in combination with ipilimumab for untreated asymptomatic brain metastases < 3.0 cm in size [13]. Their findings indicated that combination immunotherapy demonstrated a meaningful intracranial benefit (57% response rate). The extracranial benefit was also comparable with a response rate of 56%. The Anti-PD-1 Brain Collaboration (ABC) phase 2 trial is another landmark study evaluating the efficacy of combined immunotherapy (ipilimumab + nivolumab) in the treatment of MBMs [8]. The sample consisted of patients with asymptomatic and local treatment-naïve brain metastases. Nivolumab alone demonstrated a 20% intracranial response rate in 20 patients; comparatively, 36 patients receiving combination nivolumab and ipilimumab had a 46% intracranial response rate. These pivotal randomized trials support the use of systemic immunotherapy in patients with small and asymptomatic brain metastases that have since changed clinical practice. Furthermore, these results emphasize the importance of early systemic treatment and routine surveillance imaging of the brain to optimize patient outcomes.

Our findings are consistent with the result from these prospective clinical trials in a “real world” cohort of patients. Patients treated with immunotherapy agents had an improved median overall survival of 9.0 months, compared to 4.0 months in non-immunotherapy-treated patients. Controlling for covariates with prognostic value and prior treatments received, patients receiving immunotherapy had decreased overall mortality risk (HR 0.468, *p* < 0.001) and improved survival at six months and two years (*p* < 0.001; *p* = 0.04, respectively), suggesting a durable response observed in patients treated with immunotherapy. 

Notably, patients who did not receive immunotherapy in the contemporary era were older and had neurological symptoms. These features are usually associated with poorer performance status and are likely the reason for the clinical decision to forgo systemic therapy. We wonder whether imaging surveillance practices may yield higher rates of asymptomatic brain metastases detection and allow patients, who may otherwise not be a candidate for immunotherapy, to be eligible for treatment. 

### 4.3. Combination Radiation and Immunotherapy

Clinical evidence evaluating combination radiation and immunotherapy is in its early stages. Currently, this research question is currently being investigated in the ABC-X phase 2 clinical trial, where patients are randomized to nivolumab and ipilimumab with STRT versus no STRT (NCT03340129). In general, it is believed that radiation therapy has a synergistic effect with immunotherapy [26]. Proposed mechanisms for this synergy relate to radiation-induced release of tumor antigens and immune-stimulatory damage-associated molecular patterns. As a result. cytotoxic T cells can more effectively respond to antigen exposure and eliminate tumor cells [27,28]. Several retrospective studies have reported improved survival in patients receiving combination radiation and immunotherapy; however, there is evidence that the combination can lead to additional radionecrosis [25,28,29]. Therefore, risks and benefits of combination treatment must be considered. Although our sample size is limited, we report a median overall survival of 9.0 months in patients receiving immunotherapy + STRT ± WBRT compared to 5.0 months on immunotherapy + WBRT. 

Our study is limited in that it is a retrospective, single center, observational study. Our data are limited by potential biases such as treatment selection, patient preference and physician factors. Multivariable analysis was used to adjust for treatment selection bias and balance background variables; however, selection bias cannot be completely eliminated from a retrospective assessment. Some of the known prognostic factors were missing such as LDH (missing in 48 (17%) patients). Sensitivity analysis was used to ensure that replacement of missing data with minimum and maximum values did not impact statistical analyses.

## 5. Conclusions

In patients diagnosed with MBMs, contemporary use of locoregional and systemic therapy have prolonged survival. The findings from this study show “real world” data are consistent with results of pivotal clinical trials in MBMs patients and support contemporary locoregional and immunotherapy practices. Future directions should include trials to establish an optimal brain surveillance protocol in asymptomatic high-risk patients with melanoma.

## Figures and Tables

**Table 1:** Demographics and clinical characteristics of the 276 patients included.

Characteristic	Frequency (%)
Age (median, IQR)	61 (50–72.25)
Gender (male)	185 (67.0)
Year of diagnosis ≥ 2012	103(37.3)
Location of primary tumor	
Head/neck	52 (18.8)
Extremity	63 (22.8)
Palms, soles, nails	16 (5.8)
Trunk	84 (30.4)
Unknown	61 (22.1)
Stage at diagnosis	
1	54 (19.6)
2	75 (27.2)
3	72 (26.1)
4	63 (22.8)
Unknown	12 (4.3)
BRAF mutation positive (*n* = 97)	42 (43.3)
LDH (*n* = 228)	
Normal limit	93 (33.7)
Upper limit	89 (32.2)
2× UNL	11 (4.0)
3× UNL	35 (30.1)
Number of extracranial metastases	
1	14 (5.1)
2–3	158 (57.3)
4–5	93 (33.7)
≥6	11 (4.0)
Number of intracranial metastases	
1	83 (30.1)
2–4	81 (29.3)
>5	110 (39.9)
Presence of neurological symptoms	191 (69.2)
Supratentorial lesions	263 (95.3)
Infratentorial lesions	77 (27.9)
Maximal diameter, cm (median, IQR)	2.27 (1.2–3.0)
Hemorrhagic lesions	101 (36.6)
Leptomeningeal lesions	19 (6.9)

IQR—Interquartile range; LDH—lactate dehydrogenase; UNL—upper normal limit.

**Table 2:** Impact of clinical and treatment parameters on overall survival using the proportional Cox hazards model: (**a**) univariable analysis; (**b**) multivariable analysis.

(a)		
Parameter	Hazard Ratio (95% CI)	*p*-Value *
Age	1.012 (1.004–1.021)	0.005
Gender (male)	0.945 (0.730–1.223)	0.667
Stage	0.958 (0.861–1.066)	0.434
LDH	1.366 (1.240–1.504)	<0.001
Number of BM	1.493 (1.288–1.730)	<0.001
Number of extracranial sites	1.202 (1.097–1.317)	<0.001
Neurological symptoms	1.397 (1.071–1.823)	0.014
Diameter	1.026 (0.945–1.114)	0.542
Supratentorial	0.998 (0.492–2.026)	0.996
Infratentorial	1.480 (1.130–1.938)	0.004
Hemorrhagic lesions	1.241 (0.964–1.599)	0.094
Leptomeningeal involvement	1.245 (0.779–1.991)	0.360
Immunotherapy	0.429 (0.300–0.615)	<0.001
Targeted therapy	0.764 (0.467–1.252)	0.286
Chemotherapy	0.699 (0.531–0.920)	0.011
STRT	0.369 (0.280–0.487)	<0.001
WBRT	1.555 (1.100–2.198)	0.012
Full craniotomy	0.369 (0.256–0.530)	<0.001
Partial craniotomy	0.935 (0.638–1.369)	0.729
**(b)**		
**Parameter**	**Hazard Ratio (95% CI)**	*p*-Value *
Age	1.004 (0.995–1.013)	0.362
Number of BM	1.105 (0.924–1.321)	0.274
Number of extracranial sites	1.199 (1.084–1.327)	<0.001
Neurological symptoms	1.404 (1.052–1.872)	0.021
Infratentorial	1.217 (0.915–1.618)	0.177
Immunotherapy	0.467 (0.312–0.700)	<0.001
Chemotherapy	0.593 (0.442–0.796)	0.001
STRT	0.456 (0.323–0.643)	<0.001
WBRT	0.721 (0.471–1.104)	0.132
Full craniotomy	0.480 (0.308–0.750)	0.001

LDH—lactate dehydrogenase; STRT—stereotactic radiation therapy; WBRT—whole brain radiation therapy; targeted therapy includes BRAF and MEK inhibitors; immunotherapy includes CTLA-4 and PD-1 inhibitors. * *p*-value was derived using the Cox Proportional Hazards Model.

**Table 3:** Summary of treatment type by era.

Treatment	Total (273)	Pre-2012 Era (173) *	Contemporary Era (103) *	Difference (%)	*p*-Value ^†^
**Radiation therapy**					
STRT	92 (33.3%)	40 (23.1%)	52 (50.5%)	+27.4%	<0.001
WBRT	230 (83.3%)	165 (95.4%)	65 (63.1%)	−32.3%	<0.001
**Systemic therapy**					
Chemotherapy	72 (26.1%)	59 (34.1%)	13 (12.6%)	−21.5%	<0.001
Targeted therapy	18 (6.5%)	0 (0%)	18 (17.5%)	+17.5%	-
Immunotherapy	44 (15.9%)	2 (1.2%)	42 (40.8%)	+39.8%	-

STRT—stereotactic radiotherapy; WBRT—whole brain radiation therapy; targeted therapy includes BRAF and MEK inhibitors; immunotherapy includes CTLA-4 and PD-1 inhibitors. * Pre-2012 era includes years 2000–2011 (*n* = 173) and the contemporary era includes years 2012–2017 (*n* = 103). ^†^ *p*-value was derived using the Fisher’s exact test.
